# Situational Judgement Test als Unterrichtsmethode für die kritische Diskussion zu wissenschaftlicher Praxis und Fehlverhalten

**DOI:** 10.1007/s10354-020-00780-z

**Published:** 2020-10-07

**Authors:** Chantal Klemmt, Sarah König

**Affiliations:** Institut für Medizinische Lehre und Ausbildungsforschung, Universitätsklinikum Würzburg, Josef-Schneider-Str. 2, 97080 Würzburg, Deutschland

**Keywords:** Medizinische Ausbildung, Wissenschaftskompetenz, Gruppendiskussion, Formative Prüfung, Entscheidungsfindung, Medical Education, Scientific competency, Group discussion, Formative assessment, Decision making

## Abstract

Wissenschaftskompetenz ist eine Schlüsselqualifikation für jede ärztliche Tätigkeit und sollte ebenso wie die Auseinandersetzung mit Entscheidungsprozessen von Beginn an ins Medizinstudium integriert werden. Ziel der Studie war, die Themen der guten wissenschaftlichen Praxis und des wissenschaftlichen Fehlverhaltens zu vermitteln. Ferner wurde durch die methodische Intervention „Gruppendiskussion“ eine Reflexion im Kontext der wissenschaftlichen Angemessenheit herbeigeführt. Hierfür wurde der Situational Judgement Test (SJT) von den Studierenden (*N* = 743) (individuell und in der Gruppe) bearbeitet, und dessen Resultate wurden mit den Antworten von Expert/innen/en (*N* = 23) verglichen. Nach der Gruppendiskussion näherten sich die Ergebnisse in der Verteilung und Reihenfolge den Antwortmöglichkeiten der Expert/innen/en an. Jedoch tendierten die Studierenden signifikant häufiger zu jenen Antworten, die hilfesuchende, passive und verantwortungsübertragende Optionen bedeuteten. Insgesamt hat sich der SJT als didaktische Intervention bewährt. Die Studierenden setzten sich aktiv mit den Themen auseinander, eine Diskussion konnte angeregt und das eigene Verhalten kritisch reflektiert werden.

## Summary

Scientific competence and decision-making processes are key qualifications in undergraduate medical education and should be integrated into medical studies from the very beginning. The aim of the study was to convey the topics of good scientific practice and scientific misconduct. Furthermore, the methodological intervention “group discussion” enabled a productive reflection in the context of scientific appropriateness. For this purpose, the situational judgement test (SJT) was employed at different didactic activity levels of students (*N* = 743) (individually and as a group). The rating results were compared with the answers of experts (*N* = 23). Following group discussions, the answers of students shifted (distribution and order of answer options) towards those of the experts; however, students were more likely to choose response options that implied seeking help, allowing a passive stance and transferring responsibility. Overall, the SJT was a successful didactic intervention. The students actively reviewed the topics, discussed the subjects deeply and thereby critically reflected their own behavior.

Das Heranführen und die Ausbildung von Medizinstudierenden in Wissenschaftskompetenz sind für die spätere, klinische Berufsausübung von großer Bedeutung. In Deutschland wird auf Basis des Nationalen Kompetenzbasierten Lernzielkatalogs Medizin (NKLM) sowie von der deutschen Bundesregierung initiierten Reformplänen (Masterplan Medizinstudium 2020) explizit deren Integration in das medizinische Curriculum gefordert [1, 2]. Die Lernziele zur Wissenschaftskompetenz beinhalten u. a. Rahmenbedingungen und ein Grundverständnis von Wissenschaft ebenso wie die Grundlagen von guter wissenschaftlicher Praxis und das Aufzeigen von möglichem Fehlverhalten [2–4].

Die Vermittlung dessen erfordert im Medizinstudium neben klassischen Vorlesungen auch Unterrichtskonzepte, die eine aktive Auseinandersetzung mit der Thematik herbeiführen. Solche interaktiven Unterrichtsformate ermöglichen die Reflexion von Handlungs- und Begründungswissen mit Bezug zum professionellen, wissenschaftlichen Kontext. Haltungen und Meinungen von Einzelpersonen müssen identifiziert und den Ansichten einer Gruppe bzw. einem Team sowie auch der Evidenzbasierung und den rechtlichen Rahmenbedingungen gegenübergestellt werden [1]. Als Unterrichtsmaterialien dienen hierzu idealerweise Fälle bzw. Handlungsszenarien, die exemplarische Verletzungen der guten wissenschaftlichen Praxis sowie dem wissenschaftlichen Fehlverhalten veranschaulichen. In diesen fiktiven, aber realistischen Situationen empfinden die Lernenden verschiedene Beispiele nach, um sie dann zu bewerten bzw. zu interpretieren. Vor diesem Hintergrund wurden in der vorliegenden Studie Fallbeispiele für einen Situational Judgement Test (SJT) in Videoform erstellt und den Studierenden präsentiert.

SJTs stellen eine standardisierte Methode zur Situationsbeurteilung dar. Sie wurden in der Sozialpsychologie als adaptiertes Auswahlverfahren entwickelt und sind flexibel einsetzbar [5, 6]. Den Teilnehmenden werden vorgegebene Handlungsalternativen für eine Situation vorgelegt, aus denen sie sich für ein kontextspezifisches Verhalten entscheiden. SJTs basieren auf Theorien zu Behaviorismus [7] und grundlegenden Annahmen von Persönlichkeit und Handlungserfolg [8]. Im angloamerikanischen Raum werden sie vielfältig im Rahmen von Auswahlverfahren zur Vergabe von Studienplätzen in der Medizin sowie auch in der Eignungsauswahl von Ärzt/innen/en für Weiterbildungsprogramme angewandt [5, 9–13]. Dabei ist es üblich, im Rahmen des Assessments das Urteilsvermögen in studiums- bzw. arbeitsrelevanten Situation zu erfassen und dabei insbesondere persönliche Fähigkeiten, wie z. B. Sozialkompetenz oder Kritikfähigkeit, zu bewerten. An einigen deutschsprachigen Universitäten wird der SJT ebenfalls bereits eingesetzt, um in den Auswahlverfahren für die Medizinstudienplätze [14] die soziale Kompetenz der Bewerber/innen zu testen [15]. In der vorliegenden Studie wird der SJT nicht als übliches Auswahlverfahren verwendet, sondern er dient als Unterrichtsmethode. Durch den Kontextbezug in konkreten Situationen kann der SJT als didaktisches Diskussions- und Reflexionsinstrument nachvollziehbare, aber nicht eindeutige Handlungsoptionen aufzeigen und das kritische Hinterfragen der Studierenden fördern.

Ziele der vorliegenden Studie waren es, bei den Studierenden die Diskussion und Reflexion über die Auseinandersetzung und das Entscheidungsverhalten über die Themen der guten wissenschaftlichen Praxis und des wissenschaftlichen Fehlverhaltens in einem Seminar herbeizuführen. Über diesen methodischen Zugang wurde den Studierenden ein Einstieg in die inhaltliche Thematik ermöglicht. Letztlich wurde der SJT auf unterschiedlichen Ebenen (individuell und in der Gruppe) bearbeitet und diente als Grundlage, die Meinungen der Studierenden mit denen von Expert/innen/en in einer kritischen Reflexion zu vergleichen.

## Material und Methoden

### Konzeption des Seminars bzw. der Studie

Das im Wintersemester 2016/2017 eingeführte Pflichtseminar für Erstsemesterstudierende der Human- und Zahnmedizin an der Medizinischen Fakultät der Julius-Maximilians-Universität Würzburg umfasste die übergeordneten Themen gute wissenschaftliche Praxis und wissenschaftliches Fehlverhalten. Spezifischer wurden die Themen Urheberrecht, der Umgang mit geistigem Eigentum und Forschungsdaten sowie vertraulichen personenbezogenen Patient/innen/en-Daten behandelt. Neben den 2 dargestellten Hauptzielen wurden grundlegende Begriffserläuterungen zu Wissenschaft und Wissenschaftskompetenz im medizinischen Kontext und die Relevanz guter wissenschaftlicher Praxis erläutert. Diese dienten als „gemeinsame Wissensgrundlage“ und als Voraussetzung, um die oben skizzierten Ziele zu erreichen.

Die Studie erstreckte sich über 4 Semester (WS 2016/17 bis SS 2018). Der Besuch des einmalig stattfindenden Seminartermins war für die Studierenden in einer Gruppengröße von maximal 20 Teilnehmenden verpflichtend. Jedes Semester besuchten etwa 160 Studierende der Humanmedizin und 55 Studierende der Zahnmedizin das Seminar.

### Situational Judgement Test

Der SJT bildete die zentrale Unterrichtsmethodik des Seminars [6]. Während der traditionelle SJT eine Musterlösung präsentiert, dienten im vorliegenden Test die Antworten von ausgewählten medizinischen Wissenschaftler/innen/n (Expert/innen/en) als Vergleich und Diskussionsgrundlage zu den studentischen Entscheidungen.

Die formative Selbsttestung umfasste für die Studierenden 4 Kernthemen der guten wissenschaftlichen Praxis und wissenschaftlichen Fehlverhaltens (Urheberrecht, der Umgang mit geistigem Eigentum und Forschungsdaten sowie vertraulichen personenbezogenen Patient/innen/en-Daten), die in Form von Videofällen vorgestellt wurden. Dies steht im Gegensatz zur bisherigen summativen Testung des SJTs von Persönlichkeitseigenschaften mit Musterlösungen [16].

Zur Entwicklung des SJTs identifizierte eine Planungsgruppe (*N* = 5) aus Dozierenden in der Medizin typische Situationen und formulierte jeweils 5 mögliche Handlungsoptionen. Diese basierten weder auf einer Verhaltens- noch Entscheidungstheorie, sondern wurden auf Grundlage von Erfahrungswissen der Dozierenden im Sinne einer In-vivo-Beobachtung generiert. Nach einer Pilotierungsphase sowie einem kognitiven Debriefing mit Studierenden und Dozierenden wurden die Handlungsoptionen modifiziert und sprachlich angepasst.

Während des Seminars wurde der SJT mit den erstellten Fallvideos 2‑mal durchgeführt. Zunächst wurden die Studierenden um eine persönlich favorisierte Bewertung gebeten, die sie anonym auf Tablets eingaben. Anschließend fand der Test infolge einer Diskussion in Kleingruppen statt. Hierbei mussten die Studierenden gemeinsam ein Ranking der Handlungsoptionen erstellen. Als Vergleichsgrundlage dienten die Rankings der Expert/innen/en, die bereits im Vorfeld online erhoben wurden. Die Expert/innen/en erhielten eine verschriftlichte Version der Szenarien und Handlungsoptionen. In Tab. 1 sind die Fälle und Handlungsoptionen verkürzt wiedergegeben.

**Table 1:** 

Fall	Beschreibung
1. Diebstahl von geistigem Eigentum	Eine Professorin verwendet für die Erstellung eines Kongressbeitrages Textausschnitte aus einer medizinischen Doktorarbeit ohne die Erlaubnis des Autors oder Kennzeichnung dessen. Dies begründet sie damit, dass sich der Autor aus der Forschung zurückgezogen habe. Handlungsoptionen: *A Kontakt Doktorand*, da sein geistiges Eigentum betroffen ist *B Kontakt Ombudsperson*, Informationen einholen, Vorfall wird geschildert *C Forschungsrückzug* Doktorand, somit in Ordnung, die Daten zu verwenden *D Streichung* des eigenen Namens von *Autorenliste* *E* „*Nur*“ ein *Kongress*beitrag, daher ist das Vorgehen in Ordnung
2. Plagiat durch Handyfoto	Entgegen dem Hinweis, dass das Abfotografieren von Klausuren nicht gestattet sei, wird eine Klausur mit dem Handy fotografiert. Diese wird später mit der Bitte, weiterhin zu fotografieren, bei Facebook in eine Humanmedizin-Gruppe gestellt. Handlungsoptionen: *A Profit* aus *Altklausuren* ist normal, alle machen das *B* Aufruf zur Nutzung einer *Dropbox*, da diese *sicherer* ist *C Kontakt Institut* der gestellten Klausur *D Kontakt Fachschaft*, Informationen einholen *E Kontakt Studierende*, Bitte um Löschung der Klausuren
3. Datenmanipulation	Nach einem Gespräch unter Hilfskräften wird ohne Dokumentation eine Datenerhebung gelöscht. Wie vorher im Gespräch deutlich wurde, passt diese Erhebung nicht zum Rest (widersprüchliche Daten) und würde erhebliche Mehrarbeit bedeuten. „Traue keiner Statistik, die du nicht selbst gefälscht hast“ wird als Argumentation von einer Hilfskraft angebracht. Handlungsoptionen: *A* „*Traue keiner Statistik*“ stimmt, kann niemand nachvollziehen *B Verbleib in der AG*, daher wird nichts angesprochen *C Bewusstsein* für Datenmanipulation liegt vor, nächstes Mal wissenschaftlicher Arbeiten *D Kontakt Vorgesetzter*, Vorfall wird geschildert *E Kontakt Ombudsperson*, Informationen einholen, Vorfall wird geschildert
4. Schweigepflichtverletzung	Ein Patient wird aufgrund eines informellen Gespräches zwischen Studierenden in der Mensa identifiziert. Sowohl der Name des Patienten, dessen Erkrankung als auch die Auswirkungen auf sein Privatleben wurden angesprochen. Handlungsoptionen: *A Kontakt Studiendekan*, Vorfall wird geschildert *B Kontakt Studierende*, Hinweis auf Schweigepflicht *C Schweigepflicht* sollte allen Studierenden *klar*/bewusst sein *D Kontakt Lehrbeauftragten*, Bitte um Hinweis auf Schweigepflicht im Praktikum *E Kontakt ärztlicher Direktor*, Informationen einholen, Vorfall wird geschildert

#### Ranking Expert/innen/en

Im Vorfeld der Studie wurden Daten von Wissenschaftler/innen/n (Expert/innen/en) verschiedener Karrierestufen auf dem Gebiet der translationalen medizinischen Forschung (*N* = 23) eingeholt. Sie setzen sich tagtäglich mit den im Seminar behandelten Themen praktisch auseinander [17]. Potenzielle Teilnehmende wurden über Clinician-Scientist-Förderprogramme gezielt angeschrieben und um Bearbeitung der Umfrage gebeten. Diese Expert/innen/en wurden per Mail zur Teilnahme an der Online-Umfrage über eine Evaluationsplattform (EvaSys®, Lüneburg, Deutschland) eingeladen und wurden aufgefordert, ein Ranking der Handlungsoptionen im wissenschaftlichen Kontext anzufertigen.

#### Persönliche Entscheidung

Zur Durchführung des Situational Judgement Tests wurden die Videos im Seminar abgespielt. Die Studierenden wählten dann jeweils eine der 5 vordefinierten Handlungsmöglichkeiten (in Analogie von Single-Choice-Klausuraufgaben) individuell nach ihrem persönlichen Empfinden und ihrem Erfahrungsstand aus. Die Ergebnisdokumentation der individuellen Handlungsentscheidungen der Studierenden erfolgte anonymisiert und freiwillig online durch die Nutzung von Tablets (ebenfalls mittels EvaSys®). Die persönliche Entscheidung diente als Reflexionsgrundlage und erhielt in dieser Arbeit keinen weiteren Fokus.

#### Ranking Gruppendiskussion

Nach Bewertung auf Individualebene folgte eine Diskussion der Szenarien innerhalb einer Kleingruppe von maximal 5 Personen. Diese Erweiterung führte zu einem Ranking der Handlungsoptionen im wissenschaftlichen Kontext. Hierbei wurden die Situationen inklusive der Handlungsoptionen nach dem allgemeinen von den Studierenden empfundenen wissenschaftlichen Verständnis ohne das Beisein eines Moderators beurteilt. Im Unterschied zur zuvor durchgeführten Single-Choice-Wahl, wurden alle Handlungsoptionen in eine für die Studierenden sinnvolle und im wissenschaftlichen Kontext angemessene Reihenfolge gebracht. Die Ergebnisse des Rankings aus der Gruppenarbeitsphase wurden pro Gruppe stellvertretend von einer Person im Plenum vorgestellt und dem Ranking der Expert/innen/en gegenübergestellt. Hierbei ist zu betonen, dass das Ranking der Expert/innen/en nicht als richtig vorgegeben wurde, sondern den Studierenden eine weitere Diskussionsgrundlage bot.

Die Methode der Gruppendiskussion intendierte, ausgehend von einer Individualannahme der Studierenden ein Meinungsbild zu entwickeln, das den mehrheitlichen Gruppenkonsens traf [18, 19]. Die Studierenden hinterfragten und positionierten ihre Meinungen im Sinne eines „Individuums in öffentlicher Auseinandersetzung“ [12]. Dies geschah sowohl im Spannungsfeld der wissenschaftlichen Angemessenheit als auch in der „peer group“ aus Kommiliton/innen/en.

### Auswertung der Daten

Die persönliche Auswahl der Studierenden für eine Handlungsoption (Single-Choice) wurde in dieser Arbeit nicht berücksichtigt, diese diente lediglich als Diskussionsgrundlage und Voraussetzung für die Gruppendiskussion. Die Auswertung der Rankings der Gruppendiskussion und der Expert/innen/en erfolgte mit der Anwendungssoftware Excel 2016, Microsoft. Die Gewichtung der einzelnen Antworten wurde von der Chronologie des Rankings bestimmt. Die am ehesten relevante Handlungsoption erhielt den numerischen Wert von 5, die am wenigsten Relevante den numerischen Wert von 1. Die Daten wurden somit zu einem Score umgerechnet, und es wurde jeweils ein Gesamtranking erstellt, wobei die Summe der genannten relativen Häufigkeiten 100 % ergab. Die Zusammenhänge und Unterschiede zwischen den beiden Gruppen (Gruppendiskussion und Expert/innen/en) wurde mithilfe von R [20] unter Verwendung des Fisher-Tests berechnet.

### Einverständnis zur Studienteilnahme

Innerhalb der Pflichtveranstaltung des Seminars war die Teilnahme an der Studie für die Studierenden freiwillig. In diesem Fall stellten sie ihre Arbeitsergebnisse explizit für Lehrforschungszwecke zur Verfügung. Das Beantworten der SJT-Aufgaben in Einzel- und Gruppenarbeit erfolgte zu jedem Zeitpunkt anonym. Eine Beratungspflicht gemäß § 15 Berufsordnung der Ärztinnen und Ärzte lag nicht vor, da die vorliegende Studie keine biomedizinische Forschung am Menschen beinhaltete. Die datenschutzrechtlichen Bestimmungen gemäß Bayerischem Datenschutzgesetz wurden eingehalten.

## Ergebnisse

Insgesamt wurden 35 Rankings der Gruppendiskussionen, dies entsprach 743 beteiligten Studierenden, und 23 Rankings der Expert/innen/en zur Datenauswertung herangezogen. Die Expert/innen/n bestanden aus 11 Frauen und 12 Männern, von denen 21 promoviert und 9 habilitiert waren bzw. eine Professur innehatten. Das Durchschnittsalter betrug 41,8 Jahre. Die demografischen Daten der Studierenden wurden nicht im Zusammenhang mit dem SJT erhoben, jedoch können zur Charakterisierung der Grundgesamtheit der Erstsemesterstudierenden die Ergebnisse einer Studieneingangsbefragung im gleichen Seminar herangezogen werden. Von 764 Studierenden waren 493 Frauen und 264 Männer bei einem durchschnittlichen Alter von 21,1 Jahren.

**Figure 1:**
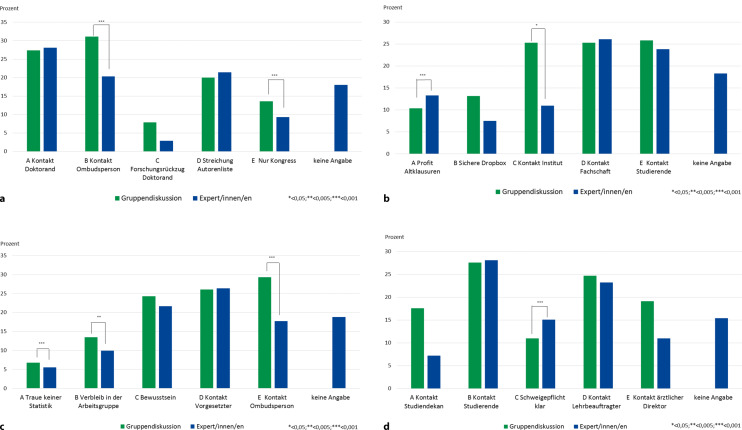

In der Abb. 1 sind die Häufigkeitsverteilungen je nach Erhebungsgruppe (Gruppendiskussion und Expert/innen/en) aufgeführt, der Signifikanz-Test verdeutlichte Unterschiede im Antwortverhalten der Teilnehmenden, die nicht zufällig waren.

Der Vergleich zwischen Gruppendiskussion und Expert/innen/en zeigte in allen Fällen, dass überwiegend Einigkeit über die „Pole“ des Rankings bestand. So wurden vornehmlich die Optionen, die am ehesten als angemessen (höchster Balken im Diagramm) und am wenigsten angemessen (niedrigster Balken im Diagramm) angesehen wurden, ohne signifikante Unterschiede beurteilt. Lediglich beim dritten Fall „Datenmanipulation“ konnte dieser Zusammenhang nicht festgestellt werden, d. h. die Studierenden und die Expert/innen/en vergaben teilweise identische Ränge bei zugleich signifikanten Unterschieden. Dennoch unterschieden sich die Gruppendiskussion und die Expert/innen/en in Bezug auf einzelne Handlungsoptionen signifikant. Hierbei wurde insbesondere die Ombudsperson unterschiedlich bewertet. Die Studierenden gaben der Ombudsperson bei der Gruppendiskussion ausschließlich den höchsten Rang, die Expert/innen/en wichen davon ab.

Ferner wählten die Studierenden in der Gruppendiskussion über alle Fälle eher Optionen, in denen sie sich passiv verhalten, in Unsicherheit verharren oder nicht aktiv handeln (Fall 1: Handlungsoption C „Forschungsrückzug“ und E „Nur Kongress“; Fall 2: A „Profit Altklausuren“; Fall 3: Handlungsoption A „Traue keiner Statistik“ und B „Verbleib in der Arbeitsgruppe“). Des Weiteren suchten die Studierenden eher Rat bei anderen Personen und Instanzen, als dies für die Expert/innen/en zutraf (Fall 1: Handlungsoption B „Kontakt Ombudsperson“; Fall 2: Handlungsoption C „Kontakt Institut“; Fall 3: Handlungsoption E „Kontakt Ombudsperson“; Fall 4: Handlungsoption A „Kontakt Studiendekan“, D „Kontakt Lehrbeauftragter“ und E „Kontakt ärztlicher Direktor“).

## Diskussion

Die vorliegende Studie setzte den SJT als formative Prüfung ein, um die Grundannahmen von Einzelpersonen und Gruppen über gute wissenschaftliche Praxis und wissenschaftliches Fehlverhalten zu diskutieren. In einem weiteren Schritt diente die Gruppendiskussion als Reflexionsmoment, um sich mit den Situationen im Kontext der wissenschaftlichen Angemessenheit kritisch auseinanderzusetzen. Die Methodik der Gruppendiskussion regte die Studierenden zur Meinungsbildung an, die durch eine angeleitete Diskussionskultur im Seminar gefestigt werden konnte. Zudem konnte diese die Studierenden ebenso auf Gruppenphänomene [21] und eine mitunter komplizierte Entscheidungsfindung im klinischen und wissenschaftlichen Alltag vorbereiten [22, 23].

Es ergaben sich über die Beispiele hinweg signifikante Unterschiede. Diese traten v. a. bei Handlungsalternativen auf, die eher passiv abwartend („Schweigepflicht klar“; „Nur Kongress“; „Verbleib in Arbeitsgruppe“), tendenziell eher wegschauend, („Schweigepflicht klar“; „Traue keiner Statistik“) oder Verantwortung abgebend („Kontakt Institut“; „Kontakt Ombudsperson“) formuliert waren. Diese Tendenz zeigte sich jedoch nicht nur bei der Gruppendiskussion, sondern auch bei den Expert/innen/en.

Im ersten (Diebstahl von geistigem Eigentum) und dritten Fallbeispiel (Datenmanipulation) stach heraus, dass der Kontakt zu einer Ombudsperson von den Studierenden in der Gruppendiskussion als erste Option genannt wurde. Die Expert/innen/en beurteilten die Vertrauenspersonen als eher weniger relevant im wissenschaftlichen Kontext. Während des Seminars kam über die möglichen Gründe für die Abweichungen in der Reihenfolge der Handlungsoptionen eine lebhafte Diskussion in Gang. Obwohl die Studierenden explizit darauf hingewiesen wurden, dass das Ranking der Expert/innen/en nur als Diskussionsgrundlage diente, wurde dieses als realitätsnah vorgegeben angesehen. Auch die Literatur sprach der Bewertung durch Expert/innen/en einen hohen Stellenwert zu, sogar vergleichbar mit einer einzelnen Validierungsstudie mit ca. 1000 Teilnehmenden [24]. Durch ihr Erfahrungswissen und ihre Expertise im Tätigkeitskontext Medizin fungierten die Expert/innen/en somit als „subject matter experts“ [16, 25].

Bis auf Grundlagen aus der (berufs)gymnasialen Oberstufe verfügten Erstsemesterstudierende über kein gesondert curricular vermitteltes Wissen. Die Beschäftigung mit den SJT-Fällen ermöglichte den Studierenden, sich einem Grenzfall im medizinisch-wissenschaftlichen Kontext sinngemäß einer kollektiven Wissensressource anzunähern und diesen auf Basis ihrer Kenntniswelt zu entschlüsseln. Für diese Intervention vorteilhaft war, dass in Abgrenzung zum klassischen SJT den Handlungsoptionen der Studierenden keine konkreten Musterlösungen gegenübergestellt wurden, sondern die Meinungen der Expert/innen/en vorgelegt wurden. Diese dienten, wie bereits ausgeführt, als Diskussionsgrundlage und galten weder als richtig noch als falsch. Weitere Vorteile des SJTs sind die nicht eindeutigen Handlungsoptionen, die von den Studierenden bewertet werden mussten und anschließend unter dem Gesichtspunkt eines Perspektivwechsels erneut diskutiert wurden. Ferner eröffneten die vorgegebenen Handlungsoptionen den Studierenden neue Perspektiven und Umgangsmöglichkeiten in den konkreten Situationen.

Abschließend ist herauszustellen, dass ein in dieser Form realisierter formativer SJT Erstsemesterstudierenden initial zu einer guten wissenschaftliche Praxis und dem Vermeiden von wissenschaftlichem Fehlverhalten verhilft und die Diskussion und Reflexion anregt. Im Hinblick auf den Anspruch einer wissenschaftlichen Ausbildung, die longitudinal im Curriculum verankert ist und frühzeitig im Studium beginnt, fördern der SJT und die Gruppendiskussion den Einstieg in die Thematik und die prompte sowie kritische Auseinandersetzung mit diesen Themen. Wie bereits expliziert, wird der Beginn der Beschäftigung mit Wissenschaftskompetenz in den ersten beiden Studienjahren eingefordert und ist longitudinal im Curriculum zu verankern [2]. Mit dem Seminar und insbesondere dem modifizierten SJT ist ein Einstieg in die Thematik „Wissenschaftskompetenz“ bereits im ersten Semester gelungen. Das Bewusstsein für gute wissenschaftliche Praxis und wissenschaftliches Fehlverhalten konnte verstärkt werden, und die Unsicherheiten und Fragen der Studierenden konnten direkt während des Seminars besprochen werden. Die rege Diskussion von wissenschaftlicher Angemessenheit und persönlichem Handeln wurde von den Studierenden genutzt. Zusätzlich haben die Erstsemesterstudierenden Anlaufstellen und Instanzen kennengelernt, an die sie sich im Bedarfsfall wenden können.

## Limitationen

Das hier angewandte Modell der Gruppendiskussion entsprach durch das Fehlen eines Moderators oder der zufälligen Gruppenbildung nicht vollständig den klassischen Standards der Methodik in Sozial- und Marktforschung [26]. Da der SJT im vorliegenden Fall ausdrücklich mit formativer Intention herangezogen wurde, war das explizite und didaktisch angeregte Gespräch in einer Studierendengruppe das zentrale Element, das eine Entscheidungsfindung anstieß und diesen Prozess evaluierbar machte. Die nach Loos und Schäffer „laboratoriumsähnliche Umgebung“ [16] in einer Gruppendiskussion kann ebenso von einer Dissonanz zwischen theoretischer Überlegung über die Ist-Situation bei den Studierenden und dem noch fernen realen Selbsthandeln geprägt sein. Überdies sind Entscheidungsheuristiken und Phänomene in Gruppen in zukünftigen Datenevaluationen stärker zu berücksichtigen, um mögliche Einflussfaktoren und Argumentationsstrukturen zu erklären.

## Schlussfolgerung

Die Verwendung des SJTs als formative Prüfung der studentischen Annahmen kreierte einerseits einen erwähnten künstlichen Entscheidungsrahmen. Zugleich zeigte sie andererseits, wie die Studierenden durch praxisnahe Handlungsbeispiele aus dem medizinischen Umfeld zu einem frühen Zeitpunkt ihrer Ausbildung in das Feld der Wissenschaftskompetenz einbezogen und zur kritischen Reflexion angeregt werden können. Sowohl die exemplarischen Situationen, die kein explizites medizinisches Wissen verlangten, als auch die alltagsnahe filmische Darstellung unterstützten dies. Zudem ist die Wahl des frühen Studienzeitpunktes ein wichtiger Beitrag für die strukturelle Transparenz der wissenschaftlichen Tätigkeit und betont deren essenzielle Bedeutung für die spätere, ärztliche Berufstätigkeit. Die Erweiterung des SJTs auf Gruppenebene strebte darüber hinaus die Herausforderung an, studentischen Gemeinkonsens über angewandte gute wissenschaftliche Praxis und den Umgang mit wissenschaftlichem Fehlverhalten herbeizuführen.

## Ausblick

Als Bestandteil eines Seminars für Erstsemesterstudierende wird der SJT in dieser Form weiterhin stattfinden. Durch die Verbindung von gemeinsamer Konsensfindung und Konfrontation mit ethischen Grenzsituationen erscheint der Einsatz dieses Formats auch in weiteren Veranstaltungen des Medizinstudiums denkbar. Ähnlich komplexe Fragestellungen, z. B. in der Palliativmedizin, Organspende oder über den Einfluss von ökonomischen Interessen, können entsprechend didaktisch umgesetzt werden. So haben die Studierenden im Sinne einer longitudinalen curricularen Verankerung wiederkehrend die Möglichkeit, über Textbuchwissen hinaus in der Gruppe und mit Input von Expert/innen/en zu diskutieren und die zugrunde liegenden Lerninhalte gebührend zu reflektieren.

## References

[1] Gesellschaft für medizinische Ausbildung (GMA) MFeVM, Vereinigung der Hochschullehrer für Zahn‑, Mund- und Kieferheilkunde (VHZMK). Nationaler Kompetenzbasierter Lernzielkatalog Medizin (NKLM): Gesellschaft für medizinische Ausbildung (GMA), Medizinischen Fakultätentag e. V. (MFT), Vereinigung der Hochschullehrer für Zahn‑, Mund- und Kieferheilkunde (VHZMK). http://www.nklm.de/kataloge/nklm/lernziel/uebersicht. Zugegriffen: 09.09.2020.

[2] Nationale Akademie der Wissenschaften Leopoldina, Medizinischer Fakultätentag (2019). Die Bedeutung von Wissenschaftlichkeit für das Medizinstudium und die Promotion.

[3] Deutsche Forschungsgemeinschaft (2013). Sicherung guter wissenschaftlicher Praxis. Sicherung Guter Wissenschaftlicher Praxis.

[4] Universität Würzburg. Richtlinien zur Sicherung guter wissenschaftlicher Praxis und für den Umgang mit wissenschaftlichem Fehlverhalten. https://www.uni-wuerzburg.de/forschung/service/gute-wissenschaftliche-praxis/. Zugegriffen: 09.09.2020.

[5] Motowidlo SJ, Dunnette MD, Carter GW (1990). An alternative selection procedure: the low-fidelity simulation. J Appl Psychol.

[6] Weekley JA, Jones C (1997). Video-based situational testing. Pers Psychol.

[7] Wernimont PF, Campbell JP (1968). Signs, samples, and criteria. J Appl Psychol.

[8] Motowidlo SJ, Hooper AC, Jackson HL (2006). Implicit policies about relations between personality traits and behavioral effectiveness in situational judgment items. J Appl Psychol.

[9] Lievens F (2013). Adjusting medical school admission: assessing interpersonal skills using situational judgement tests. Med Educ.

[10] Kerrin M, Rowett E, Lopes S (2015). The University of Nottingham: School of Veterinary Medicine & Science (SVMS): Situational Judgement Test Pilot & Operational Delivery 2014.

[11] Lopes S, Baron H, Patterson F (2015). Specialty recruitment assessment results & scoring: Psychiatry CT1 entry. Report to HEE.

[12] Roberts C, Clark T, Burgess A, Frommer M, Grant M, Mossman K (2014). The validity of a behavioural multiple-mini-interview within an assessment centre for selection into specialty training. BMC Med Educ.

[13] Roberts C, Togno JM (2011). Selection into specialist training programs: an approach from general practice. Med J Aust.

[14] Bundesvertretung der Medizinstudierenden in Deutschland e.V. (bvmd), Medizinisches Fakultätentages e.V. (MFT). Gemeinsame Stellungnahme. Vorschlag für ein neues Modell der Studierendenauswahl in der Medizin. 2017. http://webcache.googleusercontent.com/search?q=cache:k11Ee0LOJmsJ:www.mft-online.de/files/vorschlag_f__r_ein_neues_auswahlverfahren_von_mft_und_bvmd.pdf+&cd=5&hl=de&ct=clnk&gl=de&client=firefox-b. Zugegriffen: 09.09.2020.

[15] Hampe W, Hissbach J, Kadmon M (2017). Medizinstudium: Sozial kompetente Bewerber. Dtsch Arztebl Int.

[16] Marcus B (2011). Personalpsychologie. Einführung in die Arbeitsund Organisationspsychologie.

[17] König S, Rabe C, Dobbelstein M (2017). Clinician Scientists: Strukturierte Zweigleisigkeit. Dtsch Arztebl Int.

[18] Pollock F (1955). Gruppenexperiment : ein Studienbericht.

[19] Loos P, Schäffer B (2011). Das Gruppendiskussionsverfahren: theoretische grundlagen und empirische anwendung.

[20] Team RC (2014). R: a language and environment for statistical computing.

[21] Helmstaedter V, Lenarz T, Teschner M (2015). Gruppenphänomene in ärztlichen Entscheidungen – Eine Analyse unter Assistenzärzten einer HNO-Klinik. Laryngo-Rhino-Otol.

[22] Vogd W (2004). Ärztliche Entscheidungsfindung im Krankenhaus/Decision-making by hospital physicians. Z Soziol.

[23] Harendza S, Krenz I, Klinge A, Wendt U, Janneck M (2017). Implementation of a clinical reasoning course in the internal medicine trimester of the final year of undergraduate medical training and its effect on students’ case presentation and differential diagnostic skills. GMS J Med Educ.

[24] Schuler H, Kanning UP (2006). Lehrbuch der Personalpsychologie.

[25] Friedrich K, Hofmeier H (2015). Vom Aktuariat zum ganzheitlichen Produktmanagement. Change Management in Versicherungsunternehmen.

[26] Kühn T, Koschel K-V (2018). Grundlagen: Einsatz von Gruppendiskussionen in der Praxis. Gruppendiskussionen.

